# Synergistic Surface Modification of Bromocarboxylic Acid-Oleylamine Dual Ligands for Highly Stable and Luminescent CsPbBr_3_ Perovskite Nanocrystals

**DOI:** 10.3390/molecules31010127

**Published:** 2025-12-29

**Authors:** Wenjun Chen, Rui Zhang, Xiaobo Hu, Jingsheng Ma, Duna Su, Chuanli Wu, Yanqiao Xu, Xiuxun Han

**Affiliations:** 1 Institute of Optoelectronic Materials and Devices, School of Materials Science and Engineering, Jiangxi University of Science and Technology, Ganzhou 341000, China; 2 National Rare Earth Function Materials Innovation Center, Ganzhou 341100, China; 3 National Engineering Research Center for Domestic & Building Ceramics, Jingdezhen Ceramic University, Jingdezhen 333000, China

**Keywords:** perovskite nanocrystals, CsPbBr_3_, dual-ligand modification, optical properties, stability

## Abstract

The poor stability of CsPbBr_3_ perovskite nanocrystals (PNCs) caused by weak and dynamic ligand coordination severely limits their commercial applications. Herein, a dual-ligand synergistic modification strategy based on bromocarboxylic acids (BCAs) and oleylamine (OAm) was developed to mediate the surface structures and luminescent dynamics of CsPbBr_3_ PNCs. The results reveal that carboxylate groups of BCA ligands modulate crystal growth, while its terminal Br atom forms a strong coordination with exposed Pb^2+^ on the PNCs surface, which can effectively passivate lead- and bromine-related defects. The synergistic protection of OAm ligands enhances the stability of PNCs via amino-halide electrostatic interactions and steric hindrance effects. Notably, based on the relatively dense surface coating of 4-bromobutyric acid (BBA) and OAm dual-ligands, the prepared CsPbBr_3_ PNCs exhibit a high photoluminescence quantum yield (PLQY) of 85.2 ± 2.4% and remarkable storage stability, retaining 90.2 ± 1.7% of their initial PL intensity after being stored for 63 days under ambient conditions. Furthermore, a prototype white light-emitting diode (WLED) fabricated with these PNCs displays a wide color gamut covering 122.1% of the NTSC standard and a luminous efficacy of 64.6 lm/W. This work provides a facile and feasible ligand engineering strategy to obtain highly stable and emissive PNCs.

## 1. Introduction

All-inorganic cesium lead halide perovskite nanocrystals (CsPbX_3_, X = Cl, Br, I) exhibit enormous application potential in the fields of light-emitting diodes (LEDs), lasers, photodetectors, and solar cells, owing to their exceptional optoelectronic properties including high PLQY, narrow emission spectra, tunable band gaps, and high carrier mobility [1,2,3,4]. However, the ionic crystalline nature of perovskite nanocrystals (PNCs) renders them highly sensitive to environmental conditions such as moisture, oxygen, and light irradiation, leading to structural degradation and performance deterioration, which severely limits their practical applications [5,6,7,8,9]. Therefore, developing feasible synthetic approaches for high-stability and high-performance PNCs has emerged as a vital challenge.

Currently, the preparation of CsPbX_3_ NCs is divided into two mainstream routes: the hot injection method and the ligand-assisted supersaturated recrystallization (LASR) method. Actually, CsPbX_3_ PNCs with high crystallinity can be prepared using the organic-phase hot-injection method, but it has strict requirements on reaction conditions, such as high reaction temperature and inert gas protection, which increases operational complexity [10,11]. In contrast, the LASR method can induce the formation of a supersaturated system through a rapid decrease in solubility of materials, realizing the controlled nucleation and ordered growth of PNCs under room temperature [12,13], while simultaneously optimizing the surface structure and colloidal stability of PNCs via ligand modification. Recently, researchers have carried out explorations on ligand systems of the LASR method toward the controllable synthesis of PNCs. Generally, fatty acid–alkylamine ligands such as oleic acid (OA) and oleylamine (OLA) have been used to achieve uniform and emissive CsPbBr_3_ PNCs [14,15]. However, the dynamic coordination characteristics of OA/OAm ligands often lead to ligand desorption from the PNCs surface, which subsequently leads to massive agglomeration during their purification process [16,17,18,19]. To address these challenges, Moyen incorporated the didodecyl dimethyl ammonium bromide (DDAB) in the purification of PNCs, which can effectively prevent the desorption of surface ligands and improve their PL properties [20]. Zeng et al. replaced OA with benzenesulfonic acid, in which the halide-equivalent sulfonate groups strongly chelate lead ions, realizing substantial enhancements in the optical properties and thermal stability of PNCs [21]. In addition, the LASR synthesis under the water condition exhibit the advantages of environmental friendliness and low energy consumption, however the coordination between traditional ligands and PNCs in the aqueous environment is relatively weak, making it difficult to achieve excellent dispersibility and high crystallinity, which leads to fluorescence attenuation and stability degradation of PNCs [22,23]. Actually, previous research on the LASR method for synthesizing PNCs still has several obstacles. On one hand, limited attention focuses on the synergistic regulation mechanism of ligands on the surface of PNCs. On the other hand, the internal correlation between the molecular structure of ligands and PL properties or stability of PNCs is unclear, which extremely hinders the development of a promotable and reproducible preparation method for high-quality PNCs.

In this work, we developed a modified LASR strategy for the controllable preparation of stable and efficient CsPbBr_3_ PNCs by using three dual-functional synergistic ligand systems, including BBA/OAm, 6-bromohexanoicacid (BHA)/OAm and 8-bromooctanoic acid (BOA)/OAm, respectively. Compared with sulfonated ligands that only provide sulfonate groups for Pb^2+^ chelation and single-functional halogenated ligands lacking carboxyl moieties [24,25], the -COO^−^ groups of bromocarboxylic acids can form weak coordination with Cs^+^ to precisely control crystal nucleation, and the terminal Br^−^ exhibits strong coordination with Pb^2+^ on the surface of PNCs, passivating vacancy defects and reducing non-radiative recombination centers. As a synergistic ligand, OAm builds a dense secondary coating via amino-Br^−^ electrostatic interaction and forms a steric barrier with long alkyl chains, improving the particle dispersibility and colloidal stability of PNCs. The effects of ligand structure on the PL performance and stability of PNCs are systematically clarified by varying the type of brominated carboxylic acids with different carbon chains. Significantly, the prepared CsPbBr_3_ displays bright green emission with a high PLQY of 85.2%. Meanwhile, PNCs exhibit outstanding storage stability and water resistance. Benefiting from the excellent PL properties and superb stability, CsPbBr_3_ PNCs can be successfully applied as a green conversion layer in a WLED device. This study provides a new technical route for controllable synthesis of highly stable and emissive CsPbBr_3_ PNCs toward optoelectronic application fields.

## 2. Results and Discussion

**Figure 1:**
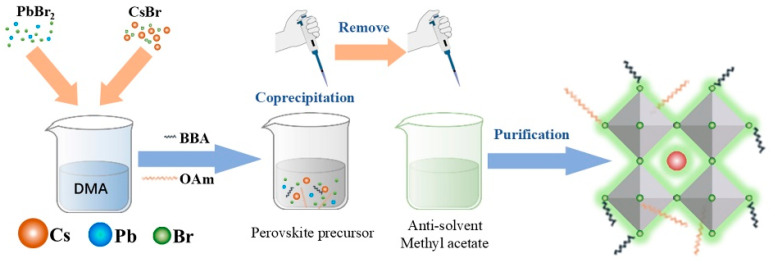
Schematic illustration of CsPbBr_3_@BBA PNCs prepared by a modified LASR method utilizing BBA and OAm as synergistic dual-ligands.

The green-emitting CsPbBr_3_ PNCs were synthesized via a modified LASR method through the synergistic modification of bromocarboxylic acid and oleylamine dual-ligands, as shown in Figure 1. Specifically, BBA can bind to unsaturated surface Pb^2+^ of PNCs via its carboxyl moiety, effectively passivating surface trap states, whereas OAm serves as a steric barrier via its hydrophobic alkyl chain, suppressing the excessive growth and particle aggregation of PNCs. Collectively, this dual-ligand system of BBA and OAm can regulate the nucleation and growth dynamics of CsPbBr_3_ PNCs throughout the preparation process. The obtained CsPbBr_3_ PNCs exhibit superior dispersibility and retain bright, stable green photoluminescence over extended storage time.

**Figure 2:**
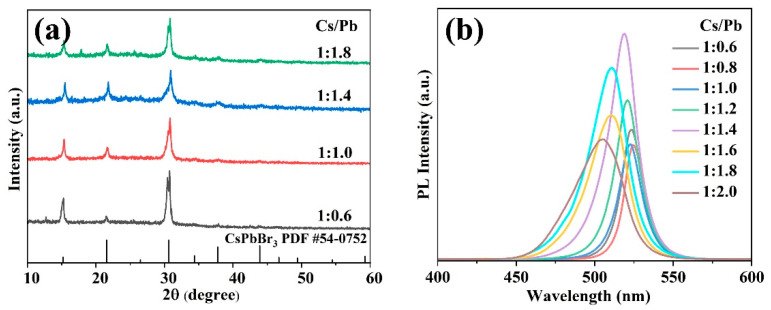
(**a**) XRD patterns and (**b**) PL spectra of CsPbBr_3_ PNCs prepared with different Cs/Pb ratios under room temperature (25 °C) and UV light (365 nm) excitation.

Figure 2a displays the XRD patterns of CsPbBr_3_ PNCs prepared with different Cs/Pb ratios by utilizing BBA and OAm as dual ligands. It was found that all the detected diffraction peaks were in good agreement with the cubic phase, matching well with the standard XRD pattern of cubic CsPbBr_3_ (PDF 54-0752). Additionally, the diffraction peaks observed at 15.1°, 21.6°, and 30.7° were indexed to the (100), (110), and (200) lattice planes of cubic CsPbBr_3_ PNCs [26,27], respectively, indicating that the CsPbBr_3_ PNCs can be synthesized via the modified LASR method with BBA/OAm dual-ligand system. The absence of some weak peaks compared with the standard PDF patterns is attributed to the nanoscale size effect, while minor additional peaks may originate from surface-adsorbed intermediate products [28]. Additionally, the intensity of the diffraction peak exhibited a gradual enhancement as the Cs/Pb ratio varied from 1:1.8 to 1:0.6, indicating that the crystallinity of PNCs was improved with the increased addition amounts of Cs precursors. The optical performance of CsPbBr_3_ PNCs prepared with different Cs/Pb ratios was explored. As illustrated in Figure 2b, the PL intensity underwent a significant enhancement as the Cs/Pb ratio decreased, attaining its maximum at a Cs/Pb ratio of 1:1.4. This phenomenon is ascribed to the formation of a more complete crystal structure in CsPbBr_3_ PNCs with reduced defect density under adequate supplement of Pb precursor, which is consistent with previous research results [13,29]. However, a sharp decrease in the PL intensity was observed when the Cs/Pb ratio was further tuned from 1:1.4 to 1:2.0. The excessive Pb^2+^ content can cause a relative deficiency of Cs^+^ monomers, which hinders the formation of well-crystallized CsPbBr_3_ PNCs, thereby escalating the density of non-radiative recombination defects and resulting in a reduction in fluorescence performance.

**Figure 3:**
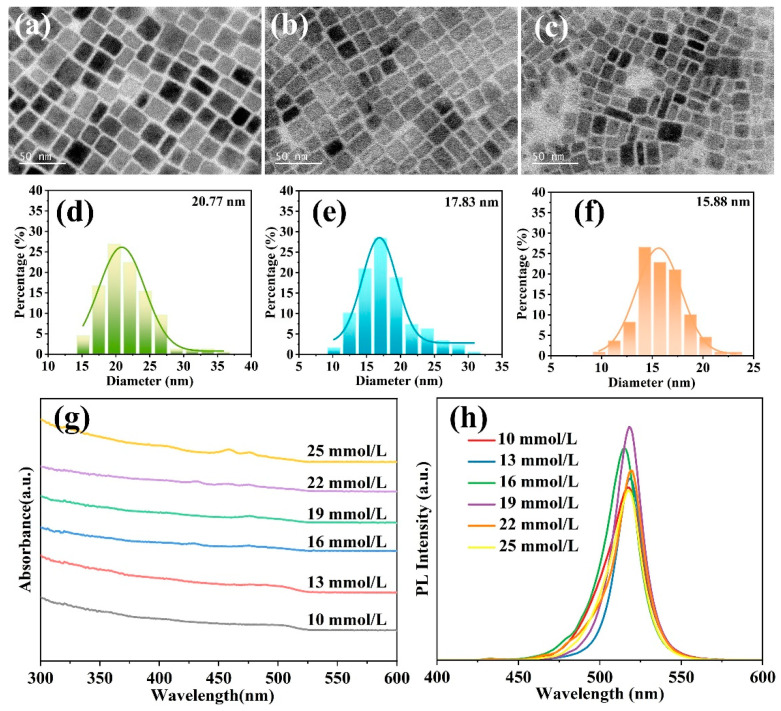
TEM images and relative size distribution histograms of CsPbBr_3_ PNCs with the BBA/OAm ligands concentration of (**a**,**d**) 13 mM, (**b**,**e**) 19 mM, and (**c**,**f**) 25 mM, respectively. (**g**) UV-vis absorption and (**h**) PL spectra of CsPbBr_3_ PNCs with different ligand concentrations.

Actually, the morphological features, chemical composition, and fluorescence behavior of PNCs are highly correlated with the ligand concentration [11,30]. As shown in Figure 3a–c, the CsPbBr_3_ PNCs prepared with different ligand concentrations exhibited a distinct cubic shape and superior dispersibility. The size distribution of PNCs in Figure 3d–f revealed that the average diameter underwent a significant reduction from 20.77 nm to 15.88 nm as the dual-ligand concentration increased from 13 mM to 25 mM, evidencing that the growth dynamics of CsPbBr_3_ PNCs can be efficiently mediated by tuning the addition of the BBA/OAm dual-ligand system. The slightly amorphous structure around the PNCs’ surfaces in TEM images is attributed to the dense organic ligand shell, which is essential for effective surface passivation and colloidal stability. In addition, the presence of dark particles on the surfaces of PNCs is associated with the formation of Pb^0^, likely due to the partial reduction of Pb^2+^ in the presence of dual-ligands [31]. Furthermore, EDS measurements were performed to determine the chemical composition of CsPbBr_3_ PNCs. As listed in Table 1, the PNCs synthesized at the dual-ligand concentration of 19 mM exhibited a Cs:Pb:Br atomic ratio of 1.00:1.15:2.96, closely approximating the ideal stoichiometric ratio (1:1:3) of CsPbBr_3_, which demonstrated the formation of high-quality CsPbBr_3_ PNCs.

The optical properties of the prepared CsPbBr_3_ PNCs were comprehensively investigated, as illustrated in Figure 3g,h. A series of absorption bands at 502~510 nm and a significant PL emission peak at 515.1~517.4 nm were observed, respectively, in good agreement with the distinctive optical characteristics of CsPbBr_3_ [32,33]. Meantime, the narrow and symmetrical emission spectra also demonstrate a relatively uniform size distribution and excellent monodispersity of PNCs. Importantly, the PL intensity of CsPbBr_3_ NCs exhibits a continuous increase with the rise of BBA/OAm ligand concentration, reaching its maximum value at 19 mM. This suggests that the dual-ligand system of BBA/OAm conducts effective modulation on the growth and luminescence kinetics of PNCs, and the obtained CsPbBr_3_ PNCs under this condition exhibit the maximum luminescent efficiency. Upon further increasing the ligand concentration to 22 and 25 mM, a notable decline in the PL intensity of PNCs was observed, arising from the strong restriction of excessive ligands on the growth process of PNCs [34,35]. This retardation limits the surface reconstruction of PNCs and concomitantly increases their surface defect density.

Actually, the molecular structure of the ligand also plays a crucial role in determining the morphology and optical properties of PNCs [36,37,38]. Therefore, this work subsequently substituted BBA with BHA and BOA, featuring extended alkyl chain segments, as shown in Figure S1. A comprehensive investigation was carried out to elucidate their mediated effects on the growth kinetics and luminescent performances of CsPbBr_3_ PNCs. As shown in Figures S2a–c and S3a–c, these CsPbBr_3_ PNCs, synthesized utilizing the dual-ligands of BHA/OAm and BOA/OAm, both displayed well-defined cubic morphology and excellent dispersibility. The size distribution results depicted in Figures S2d–f and S3d–f revealed that a notable reduction of particle size was observed when the ligand concentration increased from 13 mM to 25 mM. Specifically, the average particle diameter of BHA/OAm fabricated PNCs dropped from 14.39 nm to 10.34 nm, while that of BOA-OAm synthesized PNCs decreased from 16.36 nm to 9.67 nm. The correlation between particle size and ligand concentration is consistent with the result obtained by using BBA and OAm dual-ligands for the synthesis of PNCs. The above findings verify that the synergistic effect of bromocarboxylic acid (BBA, BHA, BOA) and fatty amine (OAm) dual-ligands can noticeably mediate the growth dynamics of CsPbBr_3_ PNCs. Significantly, the particle size of PNCs exhibited a gradual decreasing trend with the elongation of alkyl chain segments of brominated carboxylic acids, especially at the high ligand concentration. This phenomenon is mainly attributed to the intensified steric hindrance effect caused by the extended molecular chain segments of bromocarboxylic acid, which inhibits the growth process of PNCs and leads to a reduction of growth rate [39,40]. Figures S2g,h and S3g,h display the optical performances of CsPbBr_3_ PNCs with different dual-ligand concentrations of BHA/OAm and BOA/OAm. It was observed that the as-synthesized samples exhibited distinctive absorption and emission, in accordance with the characteristics of CsPbBr_3_. Additionally, the PL intensity can be effectively tuned through varying the dual-ligands concentration, establishing a critical basis for the optimization of luminescent properties of PNCs.

**Figure 4:**
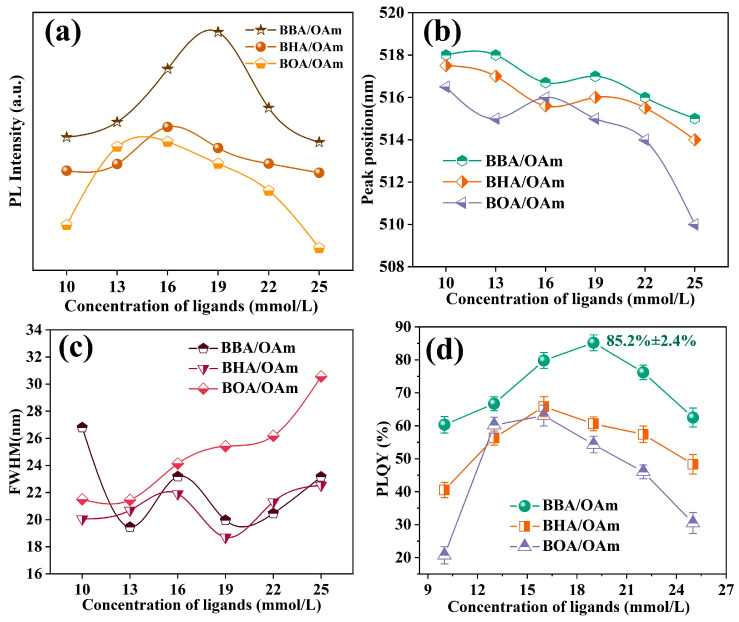
(**a**) PL intensity, (**b**) peak position, (**c**) FWHM, and (**d**) PLQY of CsPbBr_3_ PNCs with different concentrations of BBA/OAm, BHA/OAm, and BOA/OAm dual-ligands.

Figure 4 presents a comparison of the optical performances of CsPbBr_3_ PNCs synthesized using three distinct dual-ligand systems of BBA/OAm, BHA/OAm, and BOA/OAm. With the increase of the dual-ligand concentration (Figure 4a), the PL intensity of PNCs exhibits a trend of initial enhancement followed by attenuation. Notably, the BBA/OAm-prepared CsPbBr_3_ PNCs reached the maximum luminescence intensity at a ligand concentration of 19 mM. As displayed in Figure 4b, the emission peak positions of PNCs prepared using the three dual-ligand systems all showed a blue shift with the increase in ligand concentration. In combination with the results presented in Figure 3 and Figures S2 and S3, this phenomenon is mainly ascribed to the decrease in particle size caused by the increased ligand introduction, which enhances the quantum confinement effect and leads to a slight enlargement of the band gap [41]. In comparison to the BOA/OAm dual-ligands, the CsPbBr_3_ PNCs synthesized by utilizing the dual-ligand system of BBA/OAm and BHA/OAm displayed a relatively narrow full width at half maximum (FWHM) below 20 nm (Figure 4c) at the ligand concentration of 19 mM, indicating that these dual-ligands can effectively regulate the growth process of PNCs, leading to the formation of homogeneous nanoparticles. Furthermore, the PLQY values of CsPbBr_3_ PNCs prepared using the three dual-ligand systems exhibited an obvious trend of first increasing and then decreasing with the rise in ligand concentration from 10 mM to 25 mM, as seen in Figure 4d. This initial enhancement of PLQY is primarily ascribed to the efficient passivation of surface defect states by introducing the moderate amount dual-ligands, which can restrict non-radiative recombination and boost radiative recombination efficiency. However, the excessive dual-ligands may cause the crystallinity decline and lattice distortion of PNCs, resulting in the formation of additional defect states and degradation of PLQY [42]. In contrast with BHA/OAm and BOA/OAm-capped PNCs, the BBA/OAm-synthesized CsPbBr_3_ PNCs exhibited the maximum PLQY of 85.2 ± 2.4% with the ligand concentration of 19 mM. This superior optical property can be attributed to the short molecular chain and weak steric hindrance effect of BBA compared to BHA and BOA ligands, which enables dense surface ligand encapsulation and efficient surface passivation of PNCs.

**Figure 5:**
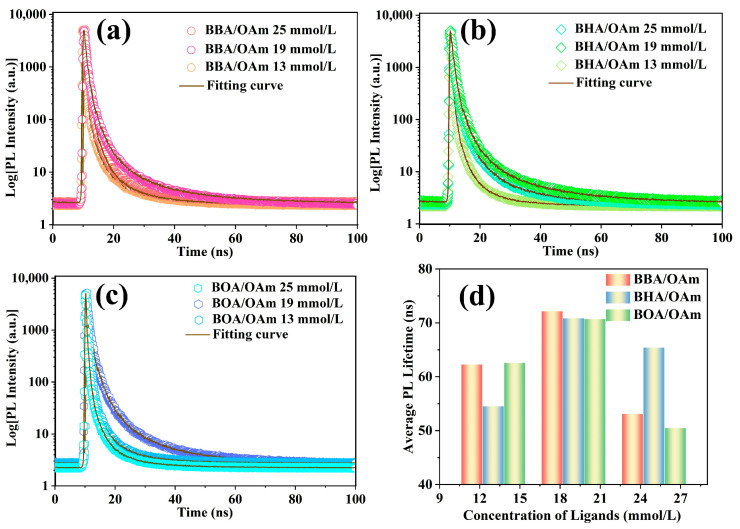
Fluorescence decay curves of CsPbBr_3_ PNCs prepared with different dual-ligand concentrations of (**a**) BBA/OAm, (**b**) BHA/OAm, and (**c**) BOA/OAm. (**d**) Average PL lifetime of NCs with different ligand concentrations.

Furthermore, time-resolved PL characterization was employed to clarify the evolution of luminescent kinetics in CsPbBr_3_ PNCs with different dual-ligand concentrations. As shown in Figure 5a–c, the PL decay curves can be well fitted by biexponential function of *I*(*t*) = *I*_0_ + *A*_1_exp(−*t*/*τ*_1_) + *A*_2_exp(−*t*/*τ*_2_), where *A*_1_ and *A*_2_ are the relative contribution of fast (*τ*_1_) and slow (*τ*_2_) decay component [32,43], then the mean PL lifetime is calculated according to the function of *τ*_avg_ = (*A*_1_*τ*_1_*^2^ + A*_2_*τ*_2_*^2^*)/(*A*_1_*τ*_1_ *+ A*_2_*τ*_2_). According to the fitting parameters summarized in Tables S1–S3 and Figure 5d, the average PL lifetime of PNCs gradually extended with the increase of ligand concentration from 13 mM to 19 mM, which was attributed to the effective passivation of surface defects by the dual-ligands, as evidenced by the significant decrease in the contribution of the short-lived component (*A*_1_). Notably, the CsPbBr_3_ PNCs capped with BBA-OAm ligands displayed the longest average lifetime of 70.91 ns, demonstrating that their superior synergistic passivation effect compared with those modified with BHA-OAm and BOA-OAm dual-ligand systems. However, the value of *A*_1_ rose noticeably as further increase in ligands concentration further increased, indicating the enhancement of non-radiative recombination at excessive ligand concentrations, which consequently reduced the mean PL lifetime. Furthermore, the evolution of PL lifetime is consistent with the trends observed in fluorescence intensity and PLQY (Figure 4). Therefore, a moderate concentration of dual-ligands is essential for achieving highly monodisperse and emissive CsPbBr_3_ PNCs.

**Figure 6:**
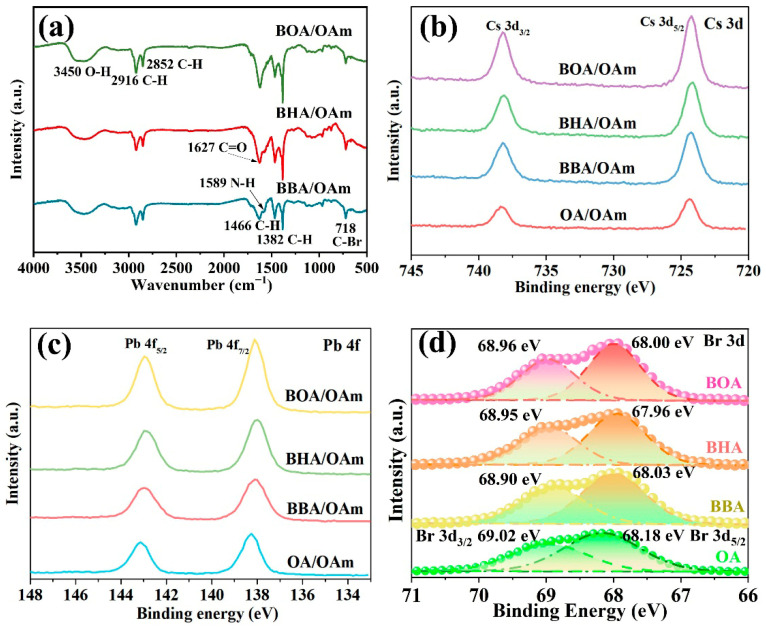
(**a**) FTIR spectra, (**b**) Cs3d, (**c**) Pb4f, and (**d**) Br 3d of CsPbBr_3_ PNCs prepared with different dual-ligands of BBA/OAm, BHA/OAm, and BOA/OAm.

Subsequently, FTIR spectra were conducted to investigate the surface coordination mechanism of CsPbBr_3_ PNCs passivated with different dual-ligand systems of BBA/OAm, BHA/OAm, and BOA/OAm. As shown in Figure 6a, a broad absorption band around 3450 cm^−1^ is attributed to the O–H stretching vibration. Peaks at 2916 cm^−1^ and 2852 cm^−1^ correspond to the asymmetric and symmetric C–H stretching vibrations of –CH_2_ and –CH_3_ groups, respectively, while those at 1466 cm^−1^ and 1382 cm^−1^ are assigned to C–H bending vibrations [44]. Additionally, the absorption peak at 1567 cm^−1^ results from the bending vibration mode of NH_2_, confirming the coordination of OAm with the PNCs [45]. The peak at 1636 cm^−1^ corresponds to the C=O stretching vibration, and the distinct peak at 718 cm^−1^ is ascribed to the C–Br stretching vibration of halogenated ligands [46], further demonstrating that bromocarboxylic acids are coordinated with the PNCs surface. Hence, both the dual-ligands of halogenated ligands (BBA, BHA, BOA) and OAm effectively coordinate with surface atoms of CsPbBr_3_ PNCs, contributing to surface defect passivation and enhancing their luminescence performance and stability.

To gain deep insights into the surface structure of PNCs synthesized with BBA/OAm, BHA/OAm, and BOA/OAm dual-ligand systems, XPS measurements were employed to determine the surface composition and element valence. The high-resolution spectra of Cs, Pb, and Br core levels are presented in Figure 6b–d. Notably, the Cs 3d core-level spectra of all three samples showed two well-resolved spin-orbit splitting peaks, including Cs 3d_3/2_ centered at 738.3 eV and Cs 3d_5/2_ at 724.4 eV, which were consistent with the binding characteristic of Cs^+^ in perovskites [12]. Similarly, the Pb 4f spectra exhibited double peaks centered near 138.7 eV (Pb 4f_7/2_) and 143.6 eV (Pb 4f_5/2_), corresponding to the valence characteristic of Pb^2+^ [17]. As shown in Figure 6d, the Br 3d spectra of all samples can be deconvoluted into two distinct spin-orbit splitting bands with the binding energy at 68.90~68.96 eV and 67.96~68.03 eV, corresponding to inner and surface Br^−^ [18], respectively. Notably, the Br 3d binding energy of the CsPbBr_3_ PNCs synthesized with BBA/OAm, BHA/OAm, and BOA/OAm dual-ligands exhibited downshifts compared with the OAm/OA modified sample. This phenomenon suggests a subtle change in the local electronic environment of Br^−^ in PNCs, which is ascribed to the altered surface coordination and electron-donating character of brominated carboxylate ligands (BBA, BHA, BOA) compared to OA. Furthermore, the relative intensity ratios between surface and inner Br^−^ of CsPbBr_3_ PNCs prepared with the dual-ligand systems of BBA/OAm, BHA/OAm and BOA/OAm are higher than that of PNCs capped with the conventional OAm/OA dual-ligands, indicating that the employment of bromocarboxylic acid ligands can effectively increase the surface Br^−^ content, thereby eliminating bromide vacancy defects of PNCs.

Based on the aforementioned investigations, we conclude that all the dual-ligand systems of BBA/OAm, BHA/OAm, and BOA/OAm exert synergistic effects on the formation and stabilization of CsPbBr_3_ PNCs. The OAm ligand can facilitate colloidal stabilization during the synthesis process of PNCs, whereas the bromocarboxylic acid can control their growth kinetics and final surface termination. The shorter ligand chain of the BBA ligand promotes dense coating and effective defect passivation, leading to superior optical stability. Thus, the dual-ligand systems collaboratively optimize surface structure, suppress non-radiative recombination, and significantly improve optical properties and stability of PNCs.

**Figure 7:**
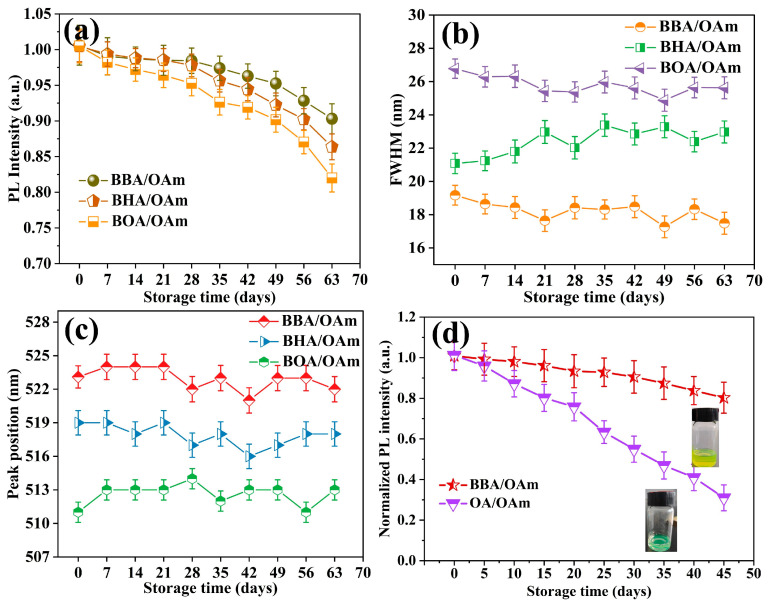
Evolution of the (**a**) PL intensity, (**b**) FWHM, and (**c**) emission peak position with storage time of CsPbBr_3_ PNCs prepared with different dual-ligands of BBA/OAm, BHA/OAm, and BOA/OAm under ambient conditions. (**d**) Water resistance stability of CsPbBr_3_ PNCs fabricated with BBA/OAm and OA/OAm.

The stability of PNCs is a critical determinant for their practical application in optoelectronic devices. Subsequently, the storage stability of CsPbBr_3_ PNCs capped with BBA/OAm, BHA/OAm, and BOA/OAm dual-ligands was systematically evaluated, respectively. The statistical robustness of the stability metrics was ensured by performing three independent synthesis and measurement cycles for each dual-ligand system. As depicted in Figure 7a–c, the PNCs synthesized by BBA/OAm dual-ligands retained 90.2 ± 1.7% of their initial PL intensity and exhibited negligible peak shift (below 2.7 ± 0.8 nm) and minimal FWHM variation (below 3.5 ± 0.6 nm) over a storage period of 63 days under ambient conditions. In contrast, the BHA/OAm and BOA/OAm-modified PNCs showed a more pronounced decline in PL intensity, remaining 86.0 ± 2.1% and 82.2 ± 2.8% of their original emissive value. The superior storage stability of BBA/OAm-capped PNCs is attributed to the shorter alkyl chain of BBA, which reduces steric hindrance and enables denser ligand coordination on the PNCs surface. The compact BBA/OAm ligand shell strengthens coordination between carboxyl/bromo functional groups and undercoordinated surface Pb^2+^ ions, effectively suppressing defect generation, halogen migration, and surface oxidation during long-term storage. The water resistance ability of CsPbBr_3_ PNCs was assessed by mixing the PNC solutions with deionized water (volume ratio 1:1) and monitoring PL performance with the prolongation of storage time under ambient laboratory conditions (room temperature 25 °C, relative humidity 50%, natural indoor lighting without UV irradiation). As shown in Figure 7d, the BBA/OAm-capped PNCs displayed superb resistance to aqueous degradation, retaining 81.5 ± 3.2% of initial emission after storing for 45 days, along with the solution exhibiting a bright-green color and no visible turbidity, whereas the PL of OA/OAm-fabricated samples was quenched rapidly, dropping below 28.1 ± 3.0% within the same period. This discrepancy arises from the ability of the ligand shell to impede water molecule penetration. In addition, the BBA/OAm-capped CsPbBr_3_ PNCs maintained the original cubic phase structure without noticeable degradation of diffraction peaks after water exposure, further confirming that they possessed excellent water resistance, as shown in Figure S4. Specifically, the dense BBA/OAm dual-ligand layer acts as an effective barrier against polar water molecules, inhibiting the decomposition of the surface structure, whereas the weakly dynamic coordination of OA/OAm dual-ligands with PNCs results in a loose ligand shell, facilitating water erosion and phase destruction of samples [5]. Overall, the CsPbBr_3_ PNCs prepared with BBA/OAm dual-ligands demonstrate significantly superior storage stability and water resistance.

**Figure 8:**
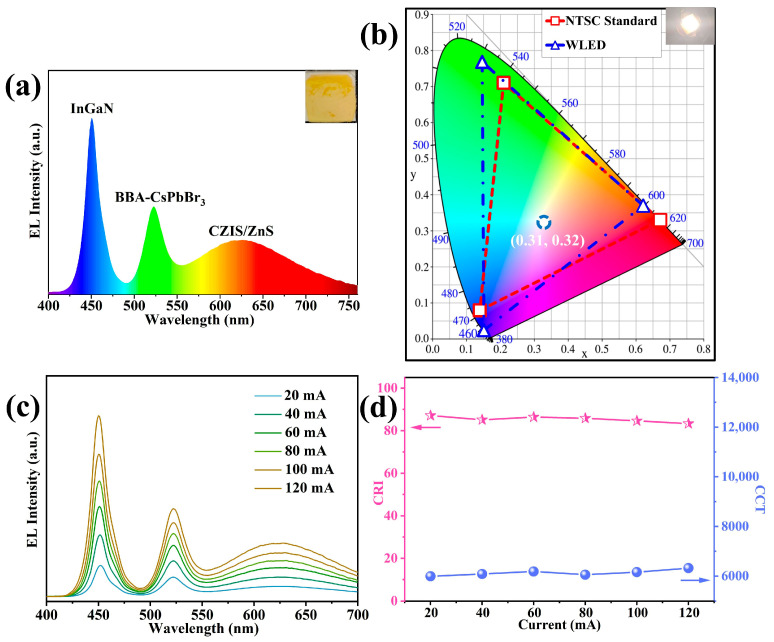
(**a**) EL spectrum of the working WLED device (inset shows a digital camera image) and (**b**) CIE coordinate and color gamut of WLED (inset displays a digital camera image under work operation) compared with the NTSC TV standard. (**c**) EL spectra, (**d**) evolution of CRI and CCT of WLED under different driven current.

To assess the application potential of CsPbBr_3_ PNCs passivated with the BBA/OAm dual-ligands in solid-state lighting, a prototype WLED was fabricated by integrating the green-emitting PNCs with a commercial red phosphor (CZIS/ZnS) on a blue LED chip (450 nm), as shown in the inset of Figure 8a,b. Under a driving current of 100 mA, the device exhibited bright white emission with CIE coordinates of (0.31, 0.32), a correlated color temperature (CCT) of 6130 K, a color rendering index (CRI) of 83.1, and a luminous efficacy of 64.6 lm/W, while its color gamut covered approximately 122.1% of the National Television System Committee (NTSC) standard. The electroluminescence (EL) intensity depicted in Figure 8c showed a continuous increase with driving current from 20 to 250 mA, along with no significant spectral shift. Notably, both the values of CRI and CCT remained stable without obvious fluctuations (Figure 8d), demonstrating excellent operational stability attributed to the effective surface passivation and suppressed ion migration provided by the dual-ligand shell. These results confirm that BBA/OAm-capped CsPbBr_3_ PNCs are promising candidates for fabricating high-performance LEDs with superior color quality, luminous efficiency and operational stability, holding great potential in solid-state lighting and backlight display fields.

## 3. Experimental

### 3.1. Materials

Cesium bromide (CsBr, AR), lead bromide (PbBr_2_, AR), 4-bromobutyric acid (BBA, 98%), 6-bromohexanoic acid (BHA, 98%), 8-bromooctanoic acid (BOA, 98%), and oleylamine (OAm, 99%) were purchased from Aladdin (Shanghai, China). N,N-Dimethylacetamide (DMA, AR), toluene (C_7_H_8_, AR), and ethyl acetate (C_4_H_8_O_2_, AR) were obtained from Sinopharm Chemical Reagent (Shanghai, China). All chemicals were used directly without any further purification.

### 3.2. Preparation of CsPbBr_3_ PNCs

In a typical synthesis of CsPbBr_3_ PNCs by the modified LARP strategy, 0.2 mmol (0.07340 g) of PbBr_2_ was dissolved in 10 mL of DMA under ambient conditions. Subsequently, 0.2 mmol (0.04256 g) of CsBr was added to the above PbBr_2_/DMA solution, followed by magnetic stirring for 10 min and ultrasonic treatment for 20 min to ensure complete dissolution. Afterward, 2.14 mmol of OAm and 0.214 mmol of BBA were introduced into the mixed solution as dual ligands. The BBA:OAm molar ratio was fixed at 1:10 based on preliminary optimization, as shown in Figure S5, ensuring effective defect passivation and colloidal stability of PNCs. The resulting mixture was magnetically stirred for 10 min to achieve a homogeneous precursor solution. Then, 0.8 mL of precursor solution was rapidly injected into 10 mL of methyl acetate (MeOAc) under vigorous stirring at room temperature (25 °C). Immediately, strong green emission of the solution could be observed under the ultraviolet irradiation (365 nm). Subsequently, the crude CsPbBr_3_ PNCs solution was centrifuged for 5 min at 2000 rpm, and the precipitate was discarded. Later, 5 mL of MeOAc was added to the supernatant, and the solution was centrifuged for 5 min at 9000 rpm. Afterwards, the precipitate was collected and redispersed in toluene. The purification process needs to be repeated twice. Finally, the CsPbBr_3_ PNCs were dispersed in toluene to obtain a clear green solution. A similar procedure was adopted for the synthesis of CsPbBr_3_ PNCs when BBA was replaced with BHA, BOA, and OA ligands.

### 3.3. Fabrication of WLED Devices

For the fabrication of the WLED device, the specific assembly process was implemented as follows: First, 50 mg of CsPbBr_3_ PNCs solution was blended with 90 mg of CZIS/ZnS red phosphors, and the mixture was subjected to continuous magnetic stirring for 10 min to guarantee homogeneous dispersion. Subsequently, an appropriate amount of curing agent was incorporated into the above mixture. The resultant composite paste was uniformly coated onto the surface of a 450 nm blue LED chip. Finally, the assembled device was placed in a 60 °C vacuum oven and cured for 4 h to obtain the target WLED device.

### 3.4. Characterizations

The crystal structure of PNCs was identified via X-ray diffraction (XRD) measurements, which were performed on a Bruker D8 Advance diffractometer (Bruker Corporation, Billerica, MA, USA) employing Cu Kα radiation with a wavelength (λ) of 0.154 nm. Morphological observations and elemental composition analysis of PNCs were conducted using a JEOL JEM-2010 (JEOL Ltd., Tokyo, Japan) transmission electron microscope (TEM) integrated with an energy dispersive spectrometer (EDS). Ultraviolet-visible (UV-vis) absorption spectra of PNCs were recorded utilizing a PerkinElmer Lambda 850 spectrophotometer (PerkinElmer Inc., Waltham, MA, USA). Photoluminescence (PL) spectra, photoluminescence quantum yield (QY), and fluorescence decay curves were collected with a Horiba Jobin Yvon Fluorolog-3 spectrometer (Horiba Jobin Yvon S.A.S., Palaiseau, France), which is equipped with a time-correlated single-photon counting (TCSPC) lifetime system and an integrating sphere for QY determination. Fourier transform infrared (FT-IR) spectra were measured on a Nicolet 6700A spectrometer (Thermo Fisher Scientific Inc., Waltham, MA, USA) within the wavenumber range of 4000–500 cm^−1^. X-ray photoelectron spectroscopy (XPS) analysis was performed using a PHI Quantera SXM spectrometer (ULVAC-PHI Inc., Chigasaki, Japan) with monochromatic Al Kα radiation to investigate the surface chemical composition and elemental valence states of the PNCs.

## 4. Conclusions

In conclusion, this study developed a modified LARP strategy for fabricating high-performance CsPbBr_3_ PNCs via the synergistic effect of bromocarboxylic acid-oleylamine dual-ligands (BBA/OAm, BHA/OAm, BOA/OAm). The OAm ligands with long alkyl chains promote colloidal stabilization of PNCs, and the brominated carboxylic ligands can regulate crystal growth and passivate lead- and bromine-related defects by terminal bromine and carboxyl groups. Notably, the dual-ligands of OAm and BBA with relatively short alkyl chains can realize the effective surface passivation due to weak steric hindrance, endowing the obtained PNCs with a high PLQY of 85.2 ± 2.4%. Importantly, the in situ passivated PNCs display exceptional stability, retaining over 90.2 ± 1.7% of initial PL intensity after being stored for 63 days under ambient conditions. Moreover, a high-performance WLED fabricated by the BBA/OAm-capped PNCs exhibits a color gamut of 122.1% NTSC, a color rendering index (CRI) of 83.1, and luminous efficacy of 64.6 lm/W. This work not only provides an effective dual-ligand modulation approach for stable and efficient PNCs but also elucidates the intrinsic relationship between ligand molecular structure and the optical properties of PNCs, establishing a theoretical guidance for the controllable synthesis of high-quality PNCs.

## Figures and Tables

**Table 1:** Relevant parameters of CsPbBr_3_ PNCs prepared with the different concentrations of BBA/OAm dual-ligands.

Ligands Concentration (mmol/L)	CsPbBr_3_ Composition	Cs/Pb/Br Ratio in Product
13	Cs_9.02_Pb_10.19_Br_25.89_	1:1.13:2.87
19	Cs_10.11_Pb_11.63_Br_29.93_	1:1.15:2.96
25	Cs_10.25_Pb_11.89_Br_31.67_	1:1.16:3.09

## Data Availability

The original contributions presented in this study are included in the article/Supplementary Materials. Further inquiries can be directed to the corresponding authors.

## Supplementary Materials

The following supporting information can be downloaded at https://www.mdpi.com/article/10.3390/molecules31010127/s1, Figure S1: Schematic diagram of molecular structure of BBA, BHA and BOA ligands; Figure S2: TEM images and relative size distribution histograms of CsPbBr_3_ PNCs with the BHA/OAm ligands concentration of (a,d) 13 mM, (b,e) 19 mM and (c,f) 25 mM, respectively. (g) UV-vis absorption and (h) PL spectra of CsPbBr_3_ PNCs with different ligands concentration of BHA/OAm; Figure S3: TEM images and relative size distribution histograms of CsPbBr_3_ PNCs with the BOA/OAm ligands concentration of (a,d) 13 mM, (b,e) 19 mM and (c,f) 25 mM, respectively. (g) UV-vis absorption and (h) PL spectra of CsPbBr_3_ PNCs with different ligands concentration of BHA/OAm; Figure S4: XRD patterns comparison of BBA/OAm-capped CsPbBr_3_ PNCs before and after water exposure for 45 days; Figure S5: PL spectra of CsPbBr_3_ PNCs with the different mole ratios of BBA/OAm dual-ligands; Table S1: Double-exponential fitting results of PL decay curves of CsPbBr_3_ PNCs synthesized with different ligand concentration of BBA/OAm; Table S2: Double-exponential fitting results of PL decay curves of CsPbBr_3_ PNCs synthesized with different ligand concentration of BHA/OAm; Table S3: Double-exponential fitting results of PL decay curves of CsPbBr_3_ PNCs synthesized with different ligand concentration of BOA/OAm.
