# Supplementary material for: Synergistic Surface Modification of Bromocarboxylic Acid-Oleylamine Dual Ligands for Highly Stable and Luminescent CsPbBr3 Perovskite Nanocrystals
Source: Molecules. 2025 Dec 29;31(1):127. doi: 10.3390/molecules31010127 (PMC12786775; doi:10.3390/molecules31010127)
Supplement: Supplementary file 1 [file molecules-31-00127-s001.zip › molecules-4076253-supplementary.pdf]

Supporting Information

Synergistic Surface Modification of Bromocarboxylic Acid-Oleylamine Dual Ligands for Highly Stable and Luminescent $CsPbBr_3$ Perovskite Nanocrystals

Wenjun Chen [1], Rui Zhang [1], Xiaobo Hu [1,2,*], Jingsheng Ma [1], Duna Su [1], Chuanli Wu [1,2], Yanqiao Xu [3,*] and Xiuxun Han [1,2,*]

1 Institute of Optoelectronic Materials and Devices, School of Materials Science and Engineering, Jiangxi University of Science and Technology, Ganzhou 341000, China; cwj20041023@163.com (W.C.); zr20050809@163.com (R.Z.); mjs20020812@163.com (J.M.); sdn20251216@163.com (D.S.); chuanli_wu@hotmail.com (C.W.)

2 National Rare Earth Function Materials Innovation Center, Ganzhou 341100, China

3 National Engineering Research Center for Domestic & Building Ceramics, Jingdezhen Ceramic University, Jingdezhen 333000, China

* Correspondence: xbhu@jxust.edu.cn (X.H.); xuyanqiao1234@163.com (Y.X.); xxhan@jxust.edu.cn (X.H.)

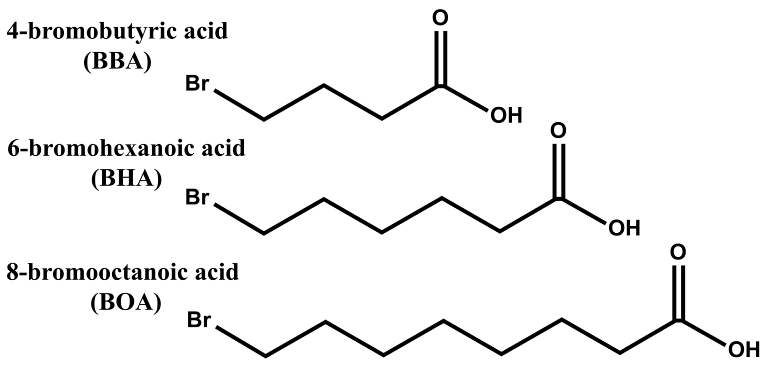

Figure S1. Schematic diagram of molecular structure of BBA, BHA and BOA ligands.

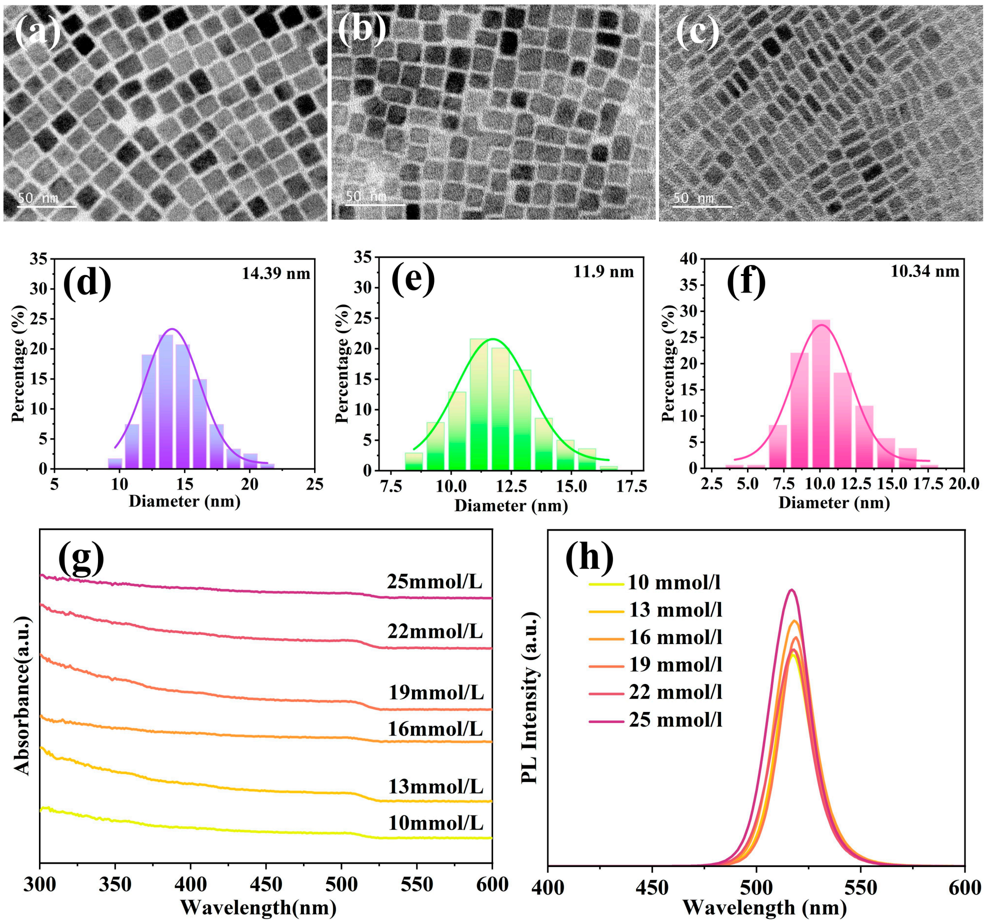

Figure S2. TEM images and relative size distribution histograms of $CsPbBr_3$ PNCs with the BHA/OAm ligands concentration of (a, d) 13 mM, (b, e) 19 mM and (c, f) 25 mM, respectively. (g) UV-vis absorption and (h) PL spectra of $CsPbBr_3$ PNCs with different ligands concentration of BHA/OAm.

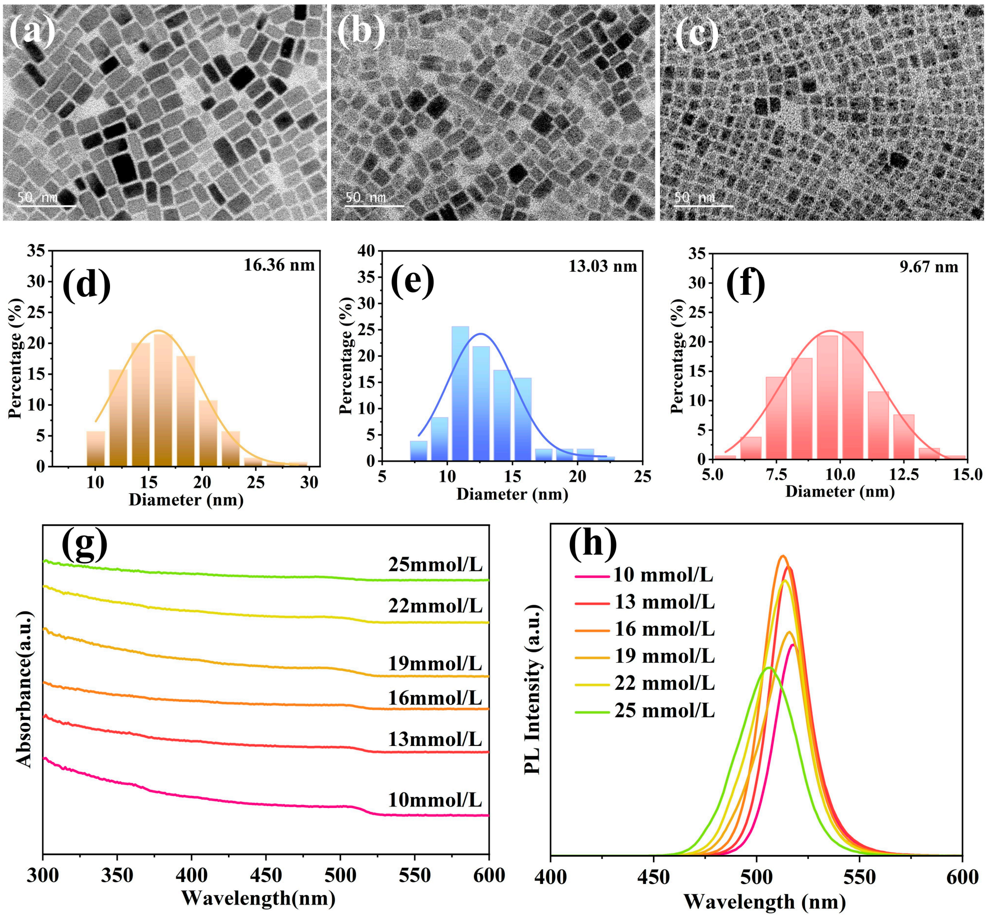

Figure S3. TEM images and relative size distribution histograms of $CsPbBr_3$ PNCs with the BOA/OAm ligands concentration of (a, d) 13 mM, (b, e) 19 mM and (c, f) 25 mM, respectively. (g) UV-vis absorption and (h) PL spectra of $CsPbBr_3$ PNCs with different ligands concentration of BHA/OAm.

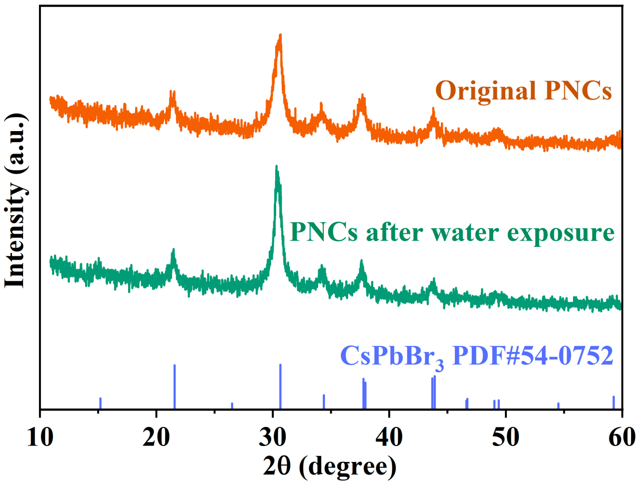

Figure S4. XRD patterns comparison of BBA/OAm-capped $CsPbBr_3$ PNCs before and after water exposure for 45 days.

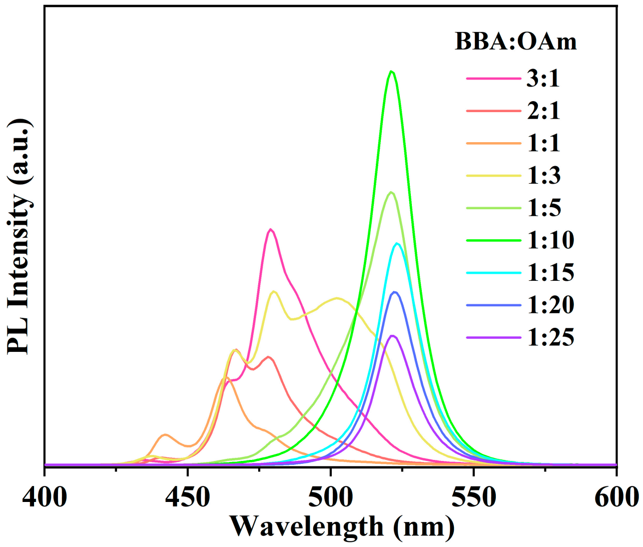

Figure S5. PL spectra of $CsPbBr_3$ PNCs with the different mole ratios of BBA/OAm dual-ligands.

Table S1. Double-exponential fitting results of PL decay curves of $CsPbBr_3$ PNCs synthesized with different ligand concentration of BBA/OAm.

Sample with different ligands concentration	τ_1 /ns	A_1/%	τ_2 /ns	A_2/%	τ_{ave} /ns
13 mmol/L	3.00	34.60	14.24	65.40	62.31
19 mmol/L	4.50	24.51	21.10	75.49	72.19
25 mmol/L	3.65	45.24	15.02	54.76	53.17

Table S2. Double-exponential fitting results of PL decay curves of $CsPbBr_3$ PNCs synthesized with different ligand concentration of BHA/OAm.

Sample with different ligands concentration	τ_1 /ns	A_1/%	τ_2 /ns	A_2/%	τ_{ave} /ns
13 mmol/L	5.00	44.43	40.00	55.57	54.56
19 mmol/L	4.25	25.56	18.76	74.44	70.91
25 mmol/L	4.35	30.60	16.87	69.40	65.44

Table S3. Double-exponential fitting results of PL decay curves of $CsPbBr_3$ PNCs synthesized with different ligand concentration of BOA/OAm.

Sample with different ligands concentration	τ_1 /ns	A_1/%	τ_2 /ns	A_2/%	τ_{ave} /ns
13 mmol/L	2.25	34.11	10.17	65.89	62.62
19 mmol/L	4.64	25.55	19.45	74.55	70.75
25 mmol/L	7.66	48.55	15.00	51.45	50.51
